# 

**Published:** 1984-01

**Authors:** 


### BRISTOL MEDICO-CHIRURGICAL JOURNAL

JANUARY 1984

VOLUME 99 (i)

NUMBER 369

